# The Role of Infection and Inflammation in the Pathogenesis of Pediatric Arterial Ischemic Stroke

**DOI:** 10.1016/j.spen.2022.100995

**Published:** 2022-09-10

**Authors:** Marie-Coralie Cornet, Charles Grose, Zinaida Vexler, Yvonne W. Wu, Heather J. Fullerton

**Affiliations:** † Department of Pediatrics, University of California San Francisco, San Francisco, California, USA.; ‡ Department of Neurology, University of California San Francisco, San Francisco, California, USA.; § Department of Pediatrics, University of Iowa, Iowa City, Iowa, USA.

## Abstract

Infections play an important role in the pathogenesis of acute ischemic stroke (AIS) in neonates and children. In neonates, chorioamnionitis or intrauterine inflammation has been implicated as a common risk factor for AIS. In infants and children, recent investigations demonstrated that even minor childhood infections are associated with subsequent increased risk for AIS. Post-infectious inflammatory mechanisms following infections with herpesviruses may lead to focal cerebral arteriopathy (FCA), one of the most common causes of AIS in a previously healthy child. Other agents such as parvovirus B19, dengue virus, and SARS-CoV-2 have recently been implicated as other potential triggers. Infections are compelling treatable stroke risk factors, with available therapies for both pathogens and downstream inflammatory effects. However, infections are common in childhood, while stroke is uncommon. The ongoing VIPS II (Vascular effects of Infection in Pediatric Stroke) study aims to identify the array of pathogens that may lead to childhood AIS and whether either unusual strains or unusual combinations of pathogens explain this paradox. Immune modulation with corticosteroids for FCA is another active area of research, with European and U.S. trials launching soon. The results of these new pediatric stroke studies combined with findings emerging from the larger field of immune-mediated post-infectious diseases will likely lead to new approaches to the prevention and treatment of pediatric stroke. This review highlights recent developments from both clinical and animal model research enhancing our understanding of this relationship between infection, inflammation, and stroke in neonates and children.

## Introduction

The COVID-19 pandemic has brought infection to the forefront for the medical community and the myriad of uncommon consequences of SARS-CoV-2 infection includes childhood arterial ischemic stroke (AIS). Even before 2020, infection was well known to play an important role in the pathogenesis of AIS in neonates and children, and multiple on-going research efforts aim to better understand this relationship. In the perinatal period, inflammation of the placenta—chorioamnionitis—increases the risk of AIS in a newborn. For childhood AIS (including the very broad age range of 29 days through 18 years), the typical “laundry list” of stroke etiologies includes several severe infections: bacterial meningitis, sepsis, and endocarditis (Table 1). However, such lists also include numerous chronic, non-infectious conditions—for example, congenital heart disease, moyamoya, sickle cell disease. Mounting evidence suggests that common childhood infections may act as stroke triggers, helping to explain why a child with a lifelong disease has a stroke at a particular point in time. However, recent evidence also suggests common viral infections may trigger strokes in otherwise healthy children presenting the paradox of a common exposure (childhood infection) associated with an uncommon outcome (childhood AIS). The immune response to *infection* likely plays an important causal role in pediatric stroke through effects on the coagulation system and arterial and cardiac endothelium. In addition, the immune response to *brain infarction* impacts both stroke severity and stroke recovery. A deeper understanding of these mechanisms has become a priority for the field because of the opportunity for intervention: the potential to use antimicrobials or immunomodulatory therapies to improve the outcomes of pediatric AIS. This review will spotlight recent developments in three areas: (1) infection as a risk factor for AIS in neonates and children; (2) the role of infection in childhood cerebral arteriopathies, in particular; and (3) the role of inflammation in the pathogenesis of brain infarction at a cellular level, with an emphasis on developmental differences between neonates and older children.

## Infection as a Risk Factor for Perinatal Arterial Ischemic Stroke

Perinatal arterial ischemic stroke (PAIS), the most common form of stroke in newborn infants, occurs in one per 2000–4000 live births.^1,2^ Infants with PAIS usually present with seizures and/or encephalopathy beginning hours to days after birth. Brain MRI typically reveals a large vessel thromboembolic infarction, most often in the left middle cerebral artery territory, though multiple areas of infarction can be seen in the setting of embolic strokes. The pathophysiology of PAIS is not well understood. It is hypothesized that the placenta releases emboli into the fetal circulation during labor and delivery and that the emboli then travel to the brain via the physiologic right-to-left shunt that results from the open foramen ovale.

Many maternal and fetal/neonatal risk factors for PAIS have been reported in large epidemiologic studies. There is strong evidence that inflammation plays a key role in the pathophysiology of PAIS. Maternal autoimmune conditions, prothrombotic disorders, and smoking or cocaine use are well-described risk factors. Placental thrombosis, prolonged rupture of membrane, and maternal chorioamnionitis or intrauterine inflammation have been implicated as common risk factors for PAIS.^3^ In a 2005 population-based study, a clinical diagnosis of chorioamnionitis in the mother was associated with a 3-fold increased risk of PAIS in the offspring.^2^ This finding was confirmed in several subsequent case-control studies.^4–6^ Maternal intrapartum fever, the clinical hallmark of chorioamnionitis, was reported in 9% of PAIS cases in a multi-national cohort study,^7^ and has been independently associated with a 10-fold increased risk of PAIS.^5^ Evidence of inflammation seen on placental examination, commonly referred to as histologic chorioamnionitis, has similarly been linked to a significantly increased risk of PAIS.^2,8,9^

When a fetus is exposed to an inflammatory intrauterine environment, it develops a fetal inflammatory response syndrome which is characterized by increased production of circulating cytokines such as IL-6 and TNF-alpha, and which can manifest as inflammatory cells seen in fetal vessels of the placenta.^10–12^ These fetal inflammatory mediators then interact with the coagulation cascade, leading to increased hypercoagulability via mechanisms such as platelet activation, injury to vascular endothelium, and impaired fibrinolysis.^3^ This interaction between inflammatory and coagulation pathways may be responsible for the increased risk of thromboembolism in the placenta and subsequent PAIS.^3,9^

Among the neonatal risk factors, bacterial meningitis and sepsis are known causes of PAIS, but only in a small minority of cases.^1,13^ Many non-infectious neonatal risk factors of PAIS are described, including genetic disorders (e.g., COL4A1 mutations) and congenital heart disease. It is notable that many infants with PAIS have both inflammatory and non-inflammatory risk factors.^2,14^

Experimental animal studies support the importance of inflammation in the pathogenesis of PAIS. In 1 rat model, the application of a prothrombotic stress to the middle cerebral artery causes a PAIS, but only if the fetus is also exposed to an inflammatory environment via maternal intraperitoneal injection of lipopolysaccharide (LPS).^15^ Although a systemic inflammatory response in the fetus could give rise to a focal arteritis in the brain,^15^ the fact that PAIS rarely recurs, and that MR angiogram rarely reveals vascular abnormalities in newborns with PAIS, argues against a focal arteritis as a major cause of PAIS in neonates. A better understanding of how perinatal inflammation leads to PAIS could lead to new strategies to prevent and treat this poorly understood condition.^1^

## Infection as a Risk Factor for Childhood Arterial Ischemic Stroke

Although severe infections like bacterial meningitis continue to cause childhood AIS, particularly in developing countries, recent investigations demonstrate a more prevalent role for minor childhood infections. A retrospective population-based study found that 40% (50/126) of childhood AIS cases (Vs 13% of 378 age-matched controls) had a medical visit for infection the month prior to stroke; of the 50 cases with preceding infection, 12 had major infections (sepsis or meningitis) while 42 had minor infections.^16^ Further analyses of the same population found that the strongest association between infection and AIS was during the 3-day period after the medical visit for infection (odds ratio [OR] 12.1, 95% CI 2.5, 57; *P*=0.002).^17^ The multicenter prospective VIPS study (Vascular effects of Infection in Pediatric Stroke) enrolled 355 cases of childhood AIS and 354 stroke-free control children (2010–2014) and confirmed a robust association between clinical infection and childhood AIS: infection in the prior week conferred a 6.5-fold risk of AIS (95% CI 3.3, 13; *P*<0.0001).^18^ The association was similar for arteriopathic, cardioembolic, and idiopathic AIS, suggesting that acute infection is a common trigger for AIS across subtypes. The most common prior infection was an upper respiratory tract infection (50%). Routine childhood vaccines were consistently protective against AIS, even after adjusting for ethnicity, geography, access to care, or socioeconomic status. Given known associations between varicella zoster virus (VZV) and stroke,^19–23^ further described below, VIPS performed immunoassays for five herpesviruses: VZV, herpes simplex virus (HSV) type 1 and 2, cytomegalovirus (CMV), and Epstein Barr virus (EBV). A case-control analysis showed that while *past* exposure to herpesviruses was similar (*P*=0.54), cases were significantly more likely than controls to have IgM antibody evidence of an *acute* herpes infection. Almost half of the cases had serologies consistent with acute herpes infection (positive IgM antibodies and/or rising IgG titers), with similar prevalence across stroke subtypes. Most were primary infections (i.e., first-time infections in seronegative children) and subclinical. HSV-1 infection was the most common. Hypothesizing that other specific pathogens might also play a role, VIPS investigators used Mass Tag-PCR to assay for 28 common childhood respiratory pathogens in plasma of a subset of the cohort (N=163) and found parvovirus B19 in 6% of cases and no controls.^24^ Seven cases had evidence of dual infection (herpesvirus and parvovirus B19), suggesting that certain *combinations* of childhood infections may increase the risk of AIS. Supporting the biologic plausibility of a link between parvovirus and AIS, parvovirus B19 infects RBCs, binds to receptors on endothelial cells, and has been linked to a wide array of vascular pathologies, including AIS in children with sickle cell disease and young adults with a cerebral arteriopathy.^25–33^

The full spectrum of pathogens that contribute to childhood stroke risk remains unknown. SARS-CoV-2 infection in children is rarely complicated by AIS,^34,35^ suggesting that this virus plays a similar role to other respiratory viruses, but rigorous data on the relative risk of AIS with different pathogens are lacking. Infection is compelling as a *treatable* stroke risk factor, with available therapies for both pathogens and downstream inflammatory effects. However, VIPS findings present a paradox: infection is common, while childhood stroke is uncommon. Possible explanations are (1) the “infection hypothesis”: unusual pathogen strains, or combinations of pathogens, lead to stroke; and (2) the “host response hypothesis”: an unusual inflammatory response to infection leads to stroke. These hypotheses will be tested by the VIPS II study which completed enrollment of another 205 cases of childhood AIS in January 2022. VIPS II will use metagenomic next generation sequencing (NGS) of serum samples and throat swabs for unbiased pathogen detection in stroke cases and controls.^36–38^ It will study gene expression profiles and inflammatory cytokine “signatures” to better understand the host response. Genetic determinants of the host response may also be important but remain challenging to study due to the difficulty obtaining adequate samples sizes for genetic analyses.

## The Role of Infection and Inflammation in Childhood Cerebral Arteriopathies

Focal cerebral arteriopathy (FCA) of childhood deserves special attention as a leading cause of childhood AIS with a presumptively post-infectious inflammatory mechanism.^39,40^ FCA typically affects school-aged children through adolescence (median age 7 years), with no sex or race predilection. Affected children, typically otherwise healthy, present with an abrupt onset hemiparesis and are found to have an acute infarct, most often in the territory of the lenticulostriate arteries. The arteriopathy is acute, monophasic, and unilateral, with irregularity or stenosis of the distal internal carotid artery (ICA) and its proximal branches.^39,40^ The hypothesized mechanism is an inflammatory response to viruses, including herpesviruses latent in the trigeminal ganglion that innervates the affected vessels.^41^ At initial presentation, children may have minimal changes on MRA. FCA then evolves rapidly, progressing over days to more severe stenosis with infarct expansion and worsening deficits. The FCA severity score (FCASS) sums disease severity (0, normal, to 4, occlusion) of 5 arterial segments.^40^ Higher FCASS correlates with larger infarct size and poorer neurological outcomes.^40,42^ Current management includes aspirin, supportive care, and presumptive treatment with high-dose corticosteroids despite the absence of efficacy data.^43^ A published Delphi consensus identified a clinical trial of corticosteroids for FCA as the highest research priority for pediatric stroke neurologists.^44^ Although herpesvirus infections like varicella may trigger the disease, pediatric virologists recommend against supplemental antiviral treatment.

**Figure 1:**
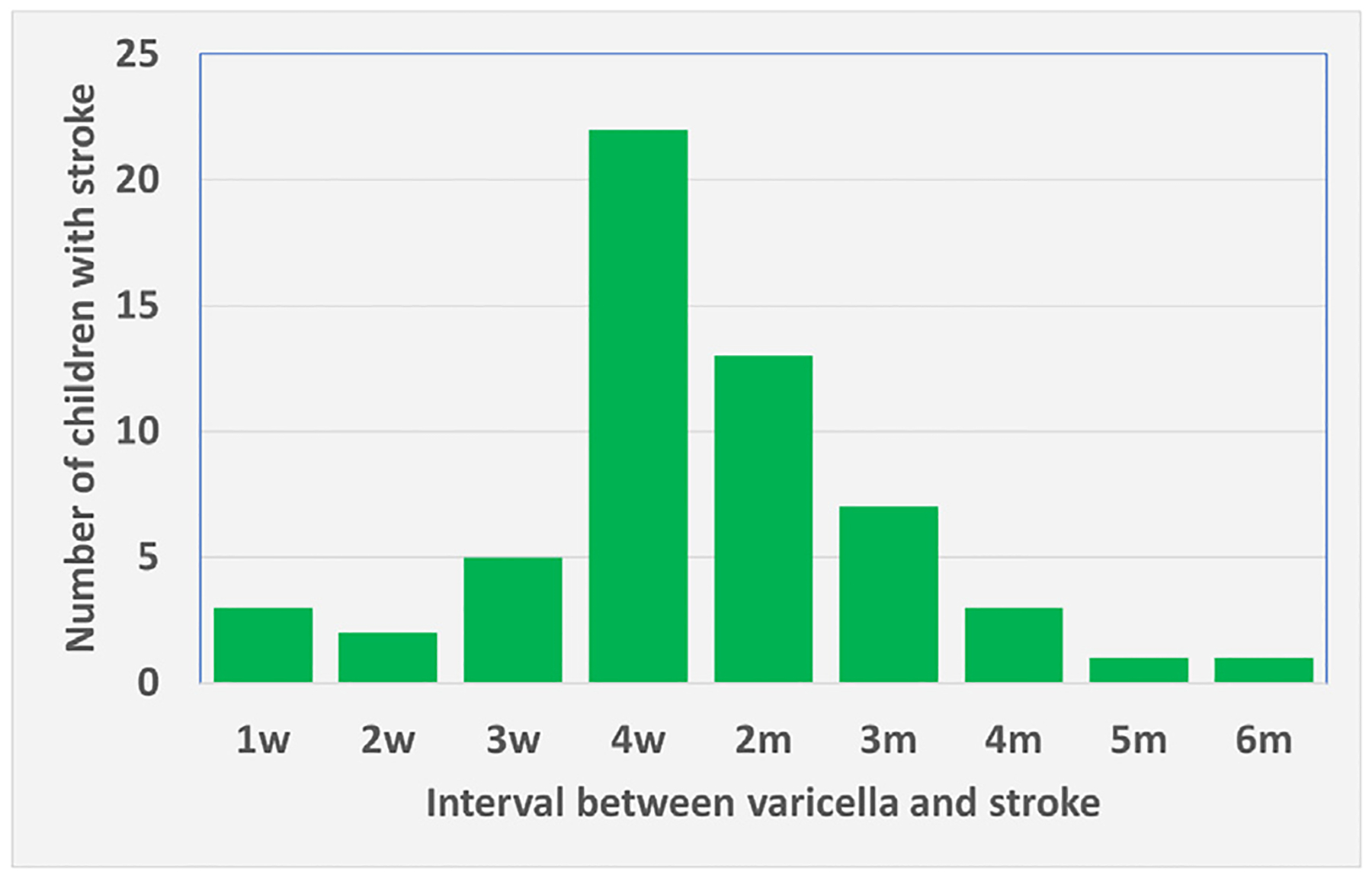
Time interval between varicella and stroke. All case reports of stroke following varicella (chickenpox) in children were tabulated. The interval between varicella and stroke was recorded in weeks (w) or months (m).

A hypothesis to account for FCA’s *unilateral and stereotypical location* involving the distal ICA is that the arteriopathy results from a pathologic immune response to viruses in the trigeminal ganglion which innervates the distal ICA and its proximal branches.^41^ Herpesviruses like VZV and HSV1 establish latency in neuronal ganglia, and can later re-activate to cause disease.^41^ The VIPS study found that 20 of 38 FCA cases had serologic evidence of a recent herpes virus infection (HSV in 18, VZV in 6; unpublished data), although positive serologies were also seen in children with other stroke etiologies.^40,45^ Older epidemiological data support a link between chicken pox (acute VZV infection, also known as varicella) and FCA.^23,46^ Histopathology is rarely available for FCA (most children survive) and existing reports are inconsistent: 1 identified VZV antigen in the affected arterial wall,^47^ while another did not.^48^ Nevertheless, these data based on numerous varicella case reports are extraordinarily compelling. In a Canadian survey, the risk of stroke after varicella in childhood was estimated to be 1:15,000.^46^ In a similar Danish survey, the risk of stroke after varicella in childhood was estimated to be 1:26,000.^49^ Since most children who lived in countries in temperate climates contracted varicella after entry into primary school, the above numbers are both supportive and meaningful. Recently all the case reports of stroke following varicella in the pediatric literature were reassessed.^41^ The interval between onset of varicella and onset of stroke was tabulated and demonstrated that most of the stroke events occurred 1 to 2 months after the bout of varicella (Fig. 1). This latent interval between varicella and stroke in children stands in juxtaposition to stroke after herpes zoster in older adults: AIS tends to occur around the same time as the herpes zoster rash.^41^

The unifying hypothesis for VZV-related stroke is that the virus originally traveled from a varicella skin vesicle on the face via sensory nerve fibers to the trigeminal ganglion. From a site of latency in the trigeminal ganglion, the virus reactivates, causing herpes zoster; it then travels anterograde via afferent fibers to the cerebral arteries, especially the distal ICA and proximal middle cerebral artery, to cause an inflammatory response.

**Figure 2:**
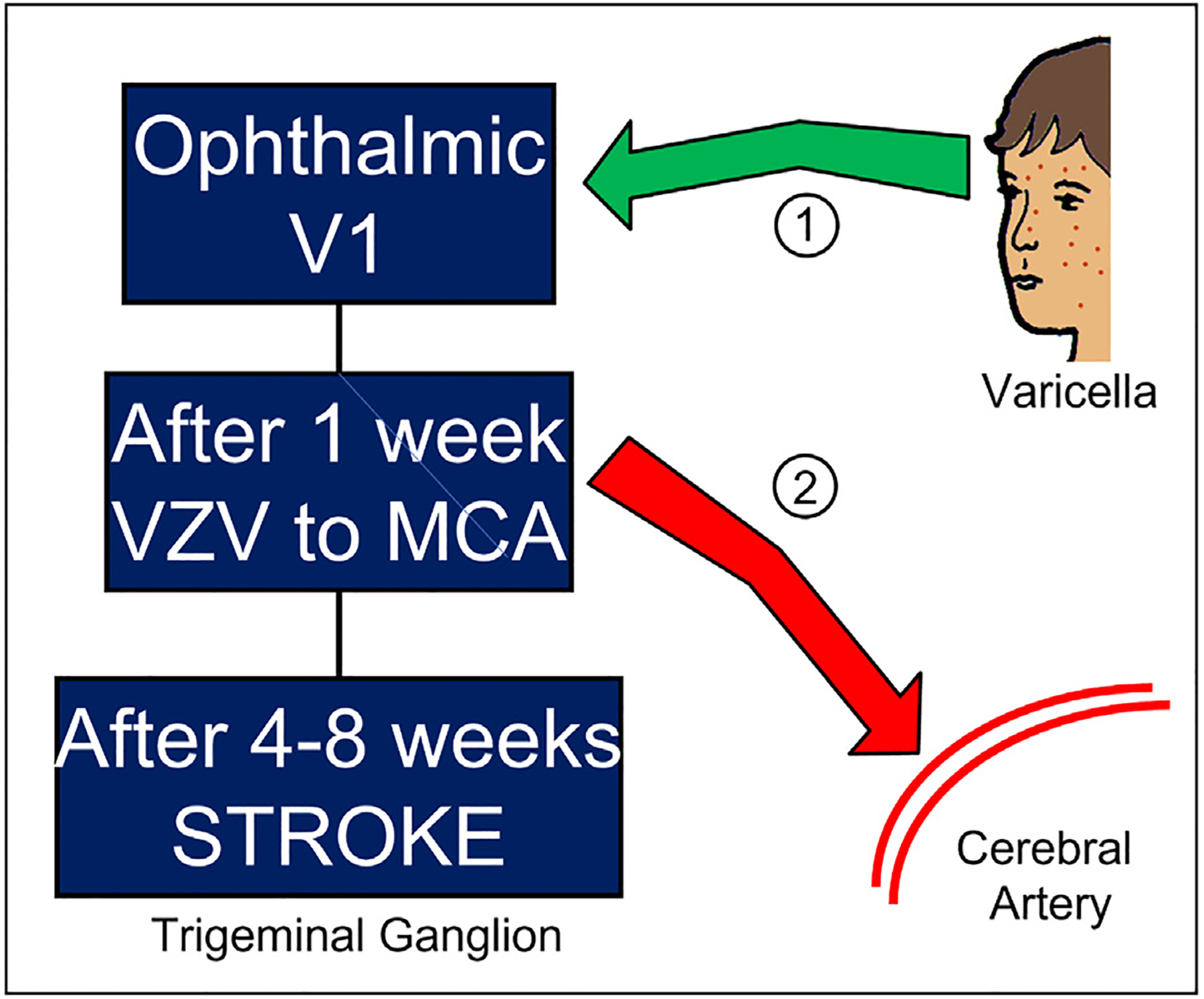
Schematic drawing to represent stroke following varicella in children. During chickenpox, varicella-zoster virus (VZV) present in the skin vesicles on the face is carried retrograde via sensory fibers into the ophthalmic branch of the trigeminal ganglion (Pathway 1). Usually, VZV then establishes a latent infection. However, on occasion VZV undergoes a replication cycle in the trigeminal ganglion within 1 week, thereby producing progeny viral particles that can travel anterograde via afferent fibers to sites innervated by the trigeminal ganglion, such as the middle cerebral artery (MCA) (Pathway 2). During a subsequent 3–7 week interval, VZV infection of the MCA induces an inflammatory response in the arterial wall that leads to stroke around 4–8 weeks after the bout of varicella.

However, very few children who develop stroke after varicella have clinical evidence of herpes zoster in the 1–2 months interval between the 2 events. For a hypothesis to explain this association, we turn to animal models for pseudorabies virus (PRV), a herpesvirus of pigs that has a similar life cycle to varicella.^50^ Studies of PRV in a rodent model have shown that virus can travel to a ganglion after primary infection and undergo another round of replication in the ganglion, in addition to establishing latency in the ganglion. Progeny virus can then enter fibers from the ganglion and be carried anterograde to other sites. Based on this round-trip model, varicella virus would travel retrograde from the face to the trigeminal ganglion during varicella, but instead of all viruses entering a latent state, some viruses would undergo another round of replication in the trigeminal ganglion, and then be carried anterograde to the cerebral arteries.^41^ All the above events would occur without clinical evidence of herpes zoster rash on the skin. Virus entering the cerebral arteries would lead to an inflammatory response (Fig. 2). The entire scenario from varicella to stroke would require 4–8 weeks; the major portion of this time period is considered to be the time required for the inflammatory response in the artery to progress to occlusion.^41^

Herpesviruses are unlikely to be the sole cause of FCA, and indeed, other pathogens have been suggested as possible causal agents: parvovirus B19, dengue virus, and SARS-CoV-2.^34,51–54^ The level of evidence is lower and hypotheses attempting to explain how those particular infections would lead to a unilateral and focal cerebral arteriopathy are lacking. Virus-induced animal models of FCA could help shed light on underlying mechanisms but such models are essentially lacking. It is possible that infection can directly alter blood-brain barrier (BBB) integrity, which, in turn, would lead to increased traffic of peripheral immune cells within the central nervous system but the magnitude and the signaling pathways may be virus-type specific.^55^ For example, to imitate viral infection in children, the first age-appropriate model for childhood arteriopathy has been recently established —by administration of the viral mimetic (and Toll-like receptor 3 (TLR3) ligand) Polyinosinic:polycytidylic acid (Poly-IC) in juvenile mice.^55^ In this juvenile mouse model, the viral mimetic disrupted endothelial tight junctions and facilitated permeability of the BBB, affecting astrocytes that form the glial limitans.^56,57^ Poly-IC rapidly enhanced the neuroinflammatory milieu and promoted neutrophil recruitment, in part by upregulation of neutrophil elastase and formation of “neurotrophil extracellular traps” (NETs).^55^ NETs protect against infection, but are also implicated in immune-mediated disease. NET formation (or “NETosis”) is induced in response to microbial infection, but must be carefully regulated to prevent excess damage to the host. Functional disturbances of the BBB, NETosis and neuroinflammation were markedly attenuated by pharmacological inhibition of neutrophil elastase.^55^ Therefore, the TLR3-neutrophil axis can play a major role in disrupting the structural-functional integrity of the BBB and distorting the developing neurovascular architecture and vascular networks. While further studies are needed to define how viral infection affects the interplay between individual cell types at the BBB and predispose the developing brain to stroke, these findings reveal a novel potential therapeutic approach to the prevention of childhood cerebral arteriopathies. It would also be important to establish models of VZV and HSV infection in juvenile animals to further understand how these particular viral infections alter neurovasculature and predispose children to stroke.

## The Role of Inflammation in the Pediatric Arterial Ischemic Stroke Pathogenesis at a Cellular Level

Emerging preclinical data demonstrates that the pathophysiology of neonatal and childhood AIS share similar mechanisms, but these diseases also have distinct molecular signatures and cellular pathways. Brain maturation is considered as a fundamental factor in immune-neurovascular interactions in stroke.^58–60^

Under physiological conditions, the expression of many proteins involved in neuronal apoptosis declines with postnatal brain maturation, along with expression of several tight junction and extracellular matrix proteins.^61^ In contrast, expression of pericyte-associated proteins increases^62^; neutrophils mature and gradually increase in their ability to recognize various inducible proteins on brain endothelium; and microglial cells, the main immune cell type in the brain, also undergo major changes during postnatal brain development.^59^

Maturation of individual components of the BBB and neurovascular unit (NVU) as a whole is not synchronized, creating “windows of susceptibility” to peripheral infection and brain inflammation in children. In fact, comparative studies in juvenile, neonatal and adult rodents subjected to various excitotoxic and inflammatory conditions (e.g., intra-cerebral injection of IL-1*β* injection or brain trauma) demonstrated more profound BBB disruption in juvenile rodents than other ages.^63,64^

To better understand mechanisms of childhood AIS, a transient middle cerebral artery occlusion (tMCAO) model in juvenile mice was recently developed.^65^ tMCAO led to more profound extravascular albumin leakage in juvenile mice^65^ than neonatal mice with a similar injury.^66^ Neutrophil accumulation was also more marked in the injured juvenile brain than in injured neonatal brain.^65,66^ To understand the involvement of peripheral leukocytes and local immune cells, microglia, to injury, signaling in microglial cells and in monocytes was genetically disrupted. Disrupted microglia-monocyte signaling reduced albumin leakage, essentially aborted monocyte infiltration, altered microglial phenotypes, and reduced injury. Furthermore, disruption of microglia-monocyte communication diminished neutrophil infiltration.^65^ These findings highlight the importance of fine details of communication between different immune cell types to injury evolvement. Literature is sparse regarding effects of childhood AIS on other brain cells but, interestingly, tMCAO in juvenile rodents led to less severe dis-myelination compared to that in adults, in part by anti-oxidative mechanisms.^67^

Animal models may also shed light on the clinical research finding that boys are more susceptible to stroke than girls and that boys have worse outcomes compared to girls with a similar injuries. Although specific mechanisms explaining sex differences in injury severity and particulars are far from being understood, a complex interplay between sex- and sex hormone-related immune activation in the brain has been shown.^68,69^ Higher susceptibility to oxidative stress of male than female rodent brain is among several identified biological processes.^70^ Distinctions in function of microglial cells in the developing male brain are thought to enhance susceptibility to stroke and neurodevelopmental diseases.^71,72^ While animal studies just scratched the surface in improving the understanding of underlying signaling mechanisms of childhood AIS, the notion of dynamic age-dependent leukocyte-endothelial interactions in modulating injury should help guide the search for effective age-appropriate therapeutic strategies.

## Conclusions and Future Directions

Infection and inflammation are important in the pathogenesis of AIS in neonates and children, with implications for potential therapies. Indeed, two clinical trials of corticosteroids for FCA—the PASTA trial (Paediatric Aspirin Steroids Arteriopathy trial) in Europe and the FOCAS trial (Focal Cerebral Arteriopathy Steroid trial) in North America—will be the firstever pediatric stroke prevention trials outside of the setting of sickle cell disease. The VIPS II study will provide new data on the array of pathogens that may trigger childhood AIS and help answer the question of whether either unusual strains or unusual combinations of pathogens explain the paradox of a common exposure associated with the rare outcome of childhood AIS. Emerging evidence on how COVID-19 severity correlates with biomarkers of interferon dysfunction (autoantibodies to interferons or mutations of interferon related genes) may also have implications for this paradox.^73,74^ Perhaps the rare child who has a stroke after a herpesvirus infection also has a dysfunction of interferons, which are the first line of defense against all viral infections. Biological samples banked in the VIPS II cohort will allow testing of such a hypothesis. We anticipate that these new pediatric stroke data combined with findings emerging from the larger field of immune-mediated post-infectious diseases will lead to new approaches to the prevention of stroke in children. In particular, a better understanding of the role of the host immune response in stroke pathogenesis, paired with targeted immune-modulating therapies, may lead to more effective interventions with fewer side effects.

## Figures and Tables

**Table 1:** An Infectious Disease “Lens” on Commonly Recognized Etiologies and Risk Factors For Childhood Arterial Ischemic Stroke. Conditions in Bold are Directly Infection-Related. Those in Italics are Chronic Conditions With Increased Stroke Risk, and Acute Infection Can Serve as a Stroke Trigger

Cardiac disease
*Congenital heart disease*
*Valvular heart disease*
Endocarditis
Myocarditis, viral
Hematologic disease
*Sickle cell disease*
*Hematologic malignancies*
*Genetic thrombophilias (e.g., Factor V Leiden)*
Acquired thrombophila from sepsis
Genetic disorders, other
*Trisomy 21*
*Neurofibromatosis type 1*
*Vascular Ehlers-Danlos*
Arteriopathies
*Moyamoya*
*PHACE syndrome*
Post-varicella arteriopathy
Vasculitis secondary to bacterial meningitis
